# Potassium penta­borate

**DOI:** 10.1107/S1600536811043807

**Published:** 2011-10-29

**Authors:** Qi Wu

**Affiliations:** a Department of Power Engineering, Xian Aeronautical College, Xian 710077, People’s Republic of China

## Abstract

The title compound, K[B_5_O_7_(OH)_2_], was obtained from a hydro­thermal reaction. The structure is composed of one K^+^ cation and a polyborate ^1^_∞_[B_5_O_7_(OH)_2_]^−^ anion, which consists of two six-membered rings linked by a common BO_4_ tetra­hedron. The [B_5_O_7_(OH)_2_]^−^ units are linked together through two exocyclic O atoms to neighbouring units, forming a helical chain structure extending parallel to [010]. Adjacent chains are further connected into a three-dimensional structure by K—O bonds and weak O—H⋯O hydrogen-bond inter­actions.

## Related literature

For the nonlinear optical properties of alkali metal borates, see: Mori *et al.* (1995 ▶). For syntheses and crystal structures in the K_2_O–B_2_O_3_–H_2_O system, see: Marezio (1969 ▶); Salentine (1987 ▶); Wang *et al.* (2006 ▶); Zhang *et al.* (2005 ▶).
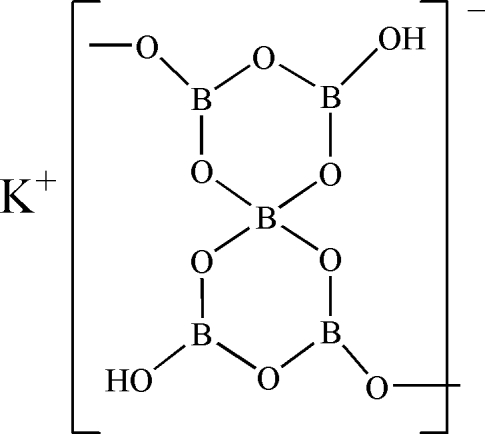


## Experimental

### 

#### Crystal data

K[B_5_O_7_(OH)_2_]
*M* *_r_* = 239.17
Monoclinic, 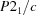

*a* = 7.6690 (3) Å
*b* = 9.0445 (3) Å
*c* = 12.2304 (4) Å
β = 119.132 (2)°
*V* = 741.01 (5) Å^3^
*Z* = 4
Mo *K*α radiation
μ = 0.74 mm^−1^
*T* = 100 K
0.14 × 0.09 × 0.07 mm

#### Data collection

Bruker APEXII diffractometer
Absorption correction: multi-scan (*SADABS*; Sheldrick, 2008*a* ▶) *T*_min_ = 0.878, *T*_max_ = 0.910
13002 measured reflections
1452 independent reflections
1343 reflections with *I* > 2σ(*I*)
*R*_int_ = 0.027

#### Refinement

*R*[*F* ^2^ > 2σ(*F* ^2^)] = 0.030
*wR*(*F* ^2^) = 0.072
*S* = 1.00
1452 reflections
136 parameters
H-atom parameters constrained
Δρ_max_ = 0.77 e Å^−3^
Δρ_min_ = −0.87 e Å^−3^

### 

Data collection: *APEX2* (Bruker, 2009 ▶); cell refinement: *SAINT* (Bruker, 2009 ▶); data reduction: *SAINT*; program(s) used to solve structure: *SHELXS97* (Sheldrick, 2008*b* ▶); program(s) used to refine structure: *SHELXL97* (Sheldrick, 2008*b* ▶); molecular graphics: *SHELXTL* (Sheldrick, 2008*b* ▶); software used to prepare material for publication: *SHELXTL*.

## Supplementary Material

Crystal structure: contains datablock(s) I, global. DOI: 10.1107/S1600536811043807/fi2113sup1.cif

Structure factors: contains datablock(s) I. DOI: 10.1107/S1600536811043807/fi2113Isup2.hkl

Additional supplementary materials:  crystallographic information; 3D view; checkCIF report

## Figures and Tables

**Table 1:** Hydrogen-bond geometry (Å, °)

*D*—H⋯*A*	*D*—H	H⋯*A*	*D*⋯*A*	*D*—H⋯*A*
O10—H10*A*⋯O6^i^	0.84	2.36	3.179 (2)	164
O12—H12*A*⋯O11^ii^	0.84	2.30	3.0346 (19)	147
O12—H12*A*⋯O4^ii^	0.84	2.50	3.170 (2)	138

Symmetry codes: (i) 
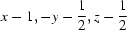
; (ii) 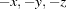.

**Fig. 1.**
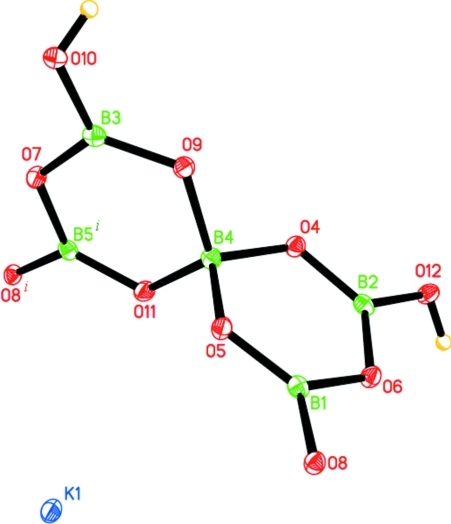
The asymmetric unit of the title compound. Displacement ellipsoids are drawn at the 50% probability level. Symmetry codes: (i) -x, -1/2 + y, -1/2 - z.

**Fig. 2.**
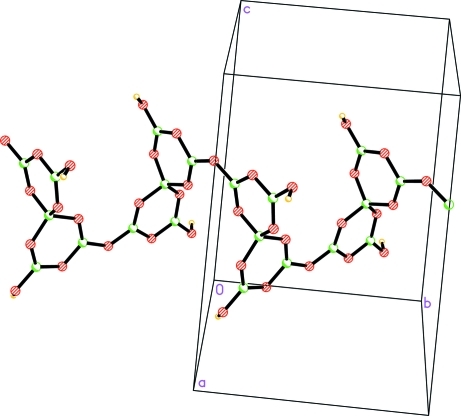
The one-dimensional chain structure constructed by [B5O7(OH)2]- units. B, O and H atoms are shown as green, red and yellow, respectively.

**Fig. 3.**
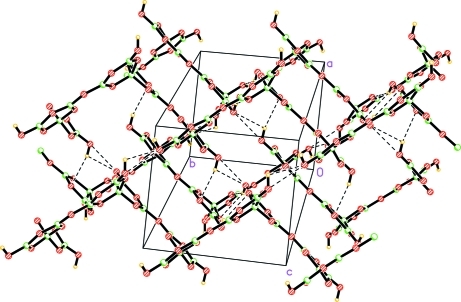
Packing view along the c axis of title compound, showing three-dimensional structure constructed by O—H···O hydrogen bonds, where all potassium cations are omitted for clarity. B, O and H atoms are shown as green, red and yellow, respectively. H bonds are drawn as dashed lines.

## supplementary crystallographic information

### Comment

Boron can form a large variety of compounds due to the variability of the coordination environment about B. In the past several decades, much interest has focused on studies of alkali metals borates because some of these compounds show interesting physical properties, such as nonlinear optical behavior for CsLiB_6_O_10_ (Mori *et al.*, 1995). So far, several phases had been obtained in the K_2_O–B_2_O_3_–H_2_O system (Marezio, 1969; Salentine, 1987; Zhang *et al.*, 2005; Wang *et al.*, 2006). In this paper, we describe the synthesis and the crystal structure of a new potassium borate, K[B_5_O_7_(OH)_2_].

It features one K^+^ cation and a ^1^_∞_[B_5_O_7_(OH)_2_]^-^ polyborate anion (Fig.1), which is closely related to the reported compound of K[B_5_O_7_(OH)_2_]^.^H_2_O (Zhang *et al.*, 2005).

The ^1^_∞_[B_5_O_7_(OH)_2_]^-^ ion consists of two six-membered rings linked by a common B atom. Each six-membered ring consists of one BO_3_ triangle, one BO_2_(OH) triangle and a common BO_4_ tetrahedron. The [B_5_O_7_(OH)_2_]^-^ units are linked via two exocyclic O atoms (O8 and O8A) to neighboring units, forming a 1-D helical chainlike structure (Fig. 2). Adjacent chains are further connected into a 3-D structure by K—O bonds and O—H···O hydrogen bonds interactions, as shown in Fig.3.

### Experimental

All reagents used in the synthesis were analytic grade and were used without further purification. A mixture of GaO(OH) (0.06 g), H_3_BO_3_(0.47 g), KNO_3_ (0.15 g) and distilled water (0.1 ml) was sealed in a Teflon-lined bomb and heated at 483 K for 3 d and then cooled to room temperature. The resulting colorless crystals were washed with hot deionized water and dried in a vacuum dryer to a constant mass at room temperature.

### Refinement

H atoms bonded to O10 and O12 atoms were positioned geometrically, and were refined riding with O—H = 0.84 Å and *U*_iso_(H) = 1.5*U*_eq_(O).

### Crystal data

**Table:**

K[B_5_O_7_(OH)_2_]	*F*(000) = 472
*M**_r_* = 239.17	*D*_x_ = 2.144 Mg m^−^^3^
Monoclinic, *P*2_1_/*c*	Mo *K*α radiation, λ = 0.71073 Å
Hall symbol: -P 2ybc	Cell parameters from 6851 reflections
*a* = 7.6690 (3) Å	θ = 3.0–30.5°
*b* = 9.0445 (3) Å	µ = 0.74 mm^−^^1^
*c* = 12.2304 (4) Å	*T* = 100 K
β = 119.132 (2)°	Rod, colourless
*V* = 741.01 (5) Å^3^	0.14 × 0.09 × 0.07 mm
*Z* = 4	

### Data collection

**Table:**

Bruker APEXII diffractometer	1452 independent reflections
Radiation source: fine-focus sealed tube	1343 reflections with *I* > 2σ(*I*)
graphite	*R*_int_ = 0.027
Detector resolution: 83.33 pixels mm^-1^	θ_max_ = 26.0°, θ_min_ = 3.0°
φ and ω scans	*h* = −9→8
Absorption correction: multi-scan (*SADABS*; Sheldrick, 2008*a*)	*k* = −11→11
*T*_min_ = 0.878, *T*_max_ = 0.910	*l* = −15→15
13002 measured reflections	

### Refinement

**Table:**

Refinement on *F*^2^	Primary atom site location: structure-invariant direct methods
Least-squares matrix: full	Secondary atom site location: difference Fourier map
*R*[*F*^2^ > 2σ(*F*^2^)] = 0.030	Hydrogen site location: inferred from neighbouring sites
*wR*(*F*^2^) = 0.072	H-atom parameters constrained
*S* = 1.00	*w* = 1/[σ^2^(*F*_o_^2^) + (0.026*P*)^2^ + 1.7263*P*] where *P* = (*F*_o_^2^ + 2*F*_c_^2^)/3
1452 reflections	(Δ/σ)_max_ < 0.001
136 parameters	Δρ_max_ = 0.77 e Å^−^^3^
0 restraints	Δρ_min_ = −0.87 e Å^−^^3^

### Special details

**Table:**

Geometry. All e.s.d.'s (except the e.s.d. in the dihedral angle between two l.s. planes) are estimated using the full covariance matrix. The cell e.s.d.'s are taken into account individually in the estimation of e.s.d.'s in distances, angles and torsion angles; correlations between e.s.d.'s in cell parameters are only used when they are defined by crystal symmetry. An approximate (isotropic) treatment of cell e.s.d.'s is used for estimating e.s.d.'s involving l.s. planes.
Refinement. Refinement of *F*^2^ against ALL reflections. The weighted *R*-factor *wR* and goodness of fit *S* are based on *F*^2^, conventional *R*-factors *R* are based on *F*, with *F* set to zero for negative *F*^2^. The threshold expression of *F*^2^ > σ(*F*^2^) is used only for calculating *R*-factors(gt) *etc*. and is not relevant to the choice of reflections for refinement. *R*-factors based on *F*^2^ are statistically about twice as large as those based on *F*, and *R*-factors based on ALL data will be even larger.

### Fractional atomic coordinates and isotropic or equivalent isotropic displacement parameters (Å^2^)

**Table:**

	*x*	*y*	*z*	*U*_iso_*/*U*_eq_	
O4	−0.2494 (2)	−0.13046 (15)	−0.14778 (13)	0.0093 (3)	
O5	−0.0757 (2)	−0.32468 (15)	−0.19460 (13)	0.0095 (3)	
O6	0.0327 (2)	−0.25360 (15)	0.01760 (12)	0.0096 (3)	
O7	−0.4011 (2)	−0.14669 (15)	−0.50951 (12)	0.0097 (3)	
O8	0.1858 (2)	−0.45303 (15)	−0.01975 (13)	0.0091 (3)	
O9	−0.4141 (2)	−0.26660 (15)	−0.33902 (12)	0.0095 (3)	
O10	−0.6325 (2)	−0.33377 (16)	−0.55281 (13)	0.0111 (3)	
H10A	−0.7360	−0.3223	−0.5465	0.017*	
O11	−0.1489 (2)	−0.08947 (15)	−0.30142 (13)	0.0101 (3)	
O12	−0.1416 (2)	−0.06260 (16)	0.05911 (13)	0.0108 (3)	
H12A	−0.0286	−0.0380	0.1172	0.016*	
B1	0.0458 (3)	−0.3419 (2)	−0.0712 (2)	0.0088 (4)	
B2	−0.1192 (3)	−0.1489 (2)	−0.0252 (2)	0.0094 (4)	
B3	−0.4815 (3)	−0.2516 (2)	−0.4635 (2)	0.0095 (4)	
B4	−0.2217 (3)	−0.2019 (2)	−0.2457 (2)	0.0093 (4)	
B5	0.2421 (3)	−0.5629 (2)	−0.0753 (2)	0.0091 (4)	
K1	0.34799 (7)	−0.07665 (5)	−0.26481 (4)	0.01448 (14)	

### Atomic displacement parameters (Å^2^)

**Table:**

	*U*^11^	*U*^22^	*U*^33^	*U*^12^	*U*^13^	*U*^23^
O4	0.0097 (7)	0.0089 (7)	0.0088 (7)	0.0014 (5)	0.0040 (6)	0.0006 (5)
O5	0.0104 (7)	0.0097 (7)	0.0085 (7)	0.0015 (5)	0.0047 (6)	0.0002 (5)
O6	0.0104 (7)	0.0098 (7)	0.0080 (7)	0.0016 (5)	0.0041 (6)	−0.0002 (5)
O7	0.0109 (7)	0.0097 (7)	0.0080 (6)	−0.0017 (6)	0.0040 (6)	−0.0001 (5)
O8	0.0102 (7)	0.0092 (7)	0.0077 (6)	0.0014 (5)	0.0043 (6)	0.0001 (5)
O9	0.0093 (7)	0.0100 (7)	0.0089 (7)	−0.0010 (5)	0.0041 (6)	0.0008 (5)
O10	0.0096 (7)	0.0126 (7)	0.0112 (7)	−0.0027 (6)	0.0051 (6)	−0.0009 (6)
O11	0.0092 (7)	0.0112 (7)	0.0085 (7)	−0.0021 (5)	0.0032 (6)	0.0009 (5)
O12	0.0098 (7)	0.0115 (7)	0.0095 (7)	0.0018 (5)	0.0035 (6)	−0.0015 (5)
B1	0.0095 (10)	0.0070 (10)	0.0105 (10)	−0.0015 (8)	0.0052 (9)	−0.0004 (8)
B2	0.0101 (10)	0.0073 (10)	0.0124 (11)	−0.0014 (8)	0.0067 (9)	0.0003 (8)
B3	0.0097 (10)	0.0074 (10)	0.0118 (11)	0.0016 (8)	0.0054 (9)	−0.0003 (8)
B4	0.0098 (10)	0.0083 (10)	0.0092 (10)	0.0000 (8)	0.0043 (9)	0.0005 (8)
B5	0.0100 (10)	0.0082 (10)	0.0111 (10)	−0.0005 (8)	0.0067 (9)	0.0000 (8)
K1	0.0143 (2)	0.0188 (3)	0.0091 (2)	0.00475 (18)	0.00474 (18)	0.00124 (17)

### Geometric parameters (Å, °)

**Table:**

O4—B2	1.347 (3)	O9—B3	1.356 (3)
O4—B4	1.464 (3)	O9—B4	1.477 (3)
O5—B1	1.342 (3)	O10—B3	1.362 (3)
O5—B4	1.482 (3)	O10—H10A	0.8400
O6—B1	1.391 (3)	O11—B5^i^	1.339 (3)
O6—B2	1.391 (3)	O11—B4	1.477 (3)
O7—B5^i^	1.381 (3)	O12—B2	1.369 (3)
O7—B3	1.391 (3)	O12—H12A	0.8400
O8—B1	1.379 (3)	B5—O11^ii^	1.339 (3)
O8—B5	1.386 (3)	B5—O7^ii^	1.381 (3)
			
B2—O4—B4	122.10 (16)	O12—B2—O6	119.56 (18)
B1—O5—B4	121.96 (16)	O9—B3—O10	123.79 (19)
B1—O6—B2	117.54 (16)	O9—B3—O7	121.38 (18)
B5^i^—O7—B3	118.31 (16)	O10—B3—O7	114.82 (17)
B1—O8—B5	131.13 (17)	O4—B4—O11	108.14 (16)
B3—O9—B4	121.29 (16)	O4—B4—O9	108.59 (16)
B3—O10—H10A	109.4	O11—B4—O9	112.07 (16)
B5^i^—O11—B4	121.94 (16)	O4—B4—O5	111.51 (16)
B2—O12—H12A	109.4	O11—B4—O5	109.41 (16)
O5—B1—O8	123.92 (19)	O9—B4—O5	107.14 (16)
O5—B1—O6	122.50 (18)	O11^ii^—B5—O7^ii^	122.64 (18)
O8—B1—O6	113.46 (17)	O11^ii^—B5—O8	123.92 (19)
O4—B2—O12	118.11 (18)	O7^ii^—B5—O8	113.41 (18)
O4—B2—O6	122.32 (18)		
			
B4—O5—B1—O8	−178.99 (18)	B2—O4—B4—O11	−103.7 (2)
B4—O5—B1—O6	5.3 (3)	B2—O4—B4—O9	134.42 (18)
B5—O8—B1—O5	3.4 (3)	B2—O4—B4—O5	16.6 (3)
B5—O8—B1—O6	179.45 (18)	B5^i^—O11—B4—O4	−125.34 (19)
B2—O6—B1—O5	3.0 (3)	B5^i^—O11—B4—O9	−5.7 (3)
B2—O6—B1—O8	−173.13 (17)	B5^i^—O11—B4—O5	113.01 (19)
B4—O4—B2—O12	171.24 (17)	B3—O9—B4—O4	135.83 (18)
B4—O4—B2—O6	−10.0 (3)	B3—O9—B4—O11	16.4 (3)
B1—O6—B2—O4	−0.7 (3)	B3—O9—B4—O5	−103.6 (2)
B1—O6—B2—O12	178.03 (17)	B1—O5—B4—O4	−14.3 (3)
B4—O9—B3—O10	165.13 (18)	B1—O5—B4—O11	105.3 (2)
B4—O9—B3—O7	−16.1 (3)	B1—O5—B4—O9	−132.97 (18)
B5^i^—O7—B3—O9	3.6 (3)	B1—O8—B5—O11^ii^	−6.9 (3)
B5^i^—O7—B3—O10	−177.49 (17)	B1—O8—B5—O7^ii^	175.04 (18)

Symmetry codes: (i) −*x*, *y*+1/2, −*z*−1/2; (ii) −*x*, *y*−1/2, −*z*−1/2.

### Hydrogen-bond geometry (Å, °)

**Table:**

*D*—H···*A*	*D*—H	H···*A*	*D*···*A*	*D*—H···*A*
O10—H10A···O6^iii^	0.84	2.36	3.179 (2)	164
O12—H12A···O11^iv^	0.84	2.30	3.0346 (19)	147
O12—H12A···O4^iv^	0.84	2.50	3.170 (2)	138

Symmetry codes: (iii) *x*−1, −*y*−1/2, *z*−1/2; (iv) −*x*, −*y*, −*z*.
